# Socio-political context as determinant of childhood maltreatment: a population-based study among women and men in East and West Germany

**DOI:** 10.1017/S2045796021000585

**Published:** 2021-11-18

**Authors:** C. Ulke, T. Fleischer, H. Muehlan, L. Altweck, S. Hahm, H. Glaesmer, J.M. Fegert, M. Zenger, H.J. Grabe, S. Schmidt, M.E. Beutel, G. Schomerus, E. Brähler, S. Speerforck

**Affiliations:** 1 Department of Psychiatry and Psychotherapy, University of Leipzig Medical Center, Leipzig, Germany; 2 Department of Health & Prevention, Institute of Psychology, University of Greifswald, Greifswald, Germany; 3 Department of Medical Psychology and Medical Sociology, University of Leipzig, Leipzig, Germany; 4 Department for Child and Adolescent Psychiatry and Psychotherapy, University Hospital Ulm, Ulm, Germany; 5 Faculty of Applied Human Studies, University of Applied Sciences Magdeburg and Stendal, Stendal, Germany; 6 Department of Psychiatry and Psychotherapy, University Medicine Greifswald, Greifswald, Germany; 7 Department of Psychosomatic Medicine and Psychotherapy, University Medical Center of the Johannes Gutenberg University Mainz, Mainz, Germany

**Keywords:** Child abuse, child neglect, child trauma, epidemiology, socio-political context

## Abstract

**Aim:**

Being subjected to childhood maltreatment has devastating long-term adverse effects and is a major risk factor for mental health problems in adult life. There is empirical support that socio-political factors can be risk factors for childhood maltreatment. Here we examine whether socio-political context predicts self-rated childhood maltreatment in a sample of the German population.

**Methods:**

Data were drawn from surveys of representative samples of the East and West German population, including participants born 1980 or earlier (5836 participants; 3146 women). Childhood maltreatment was assessed using the Childhood Trauma Screener, the German short screening version of the childhood trauma questionnaire. To examine whether socio-political context is a predictor of childhood maltreatment in women and men, we conducted logistic regression analyses.

**Results:**

We found that in women, socio-political context (West Germany) significantly predicted childhood maltreatment (odds ratio [95% confidence interval] 1.7 [1.2–2.5], 1.6 [1.1–2.3], 2.5 [1.6–4.1] and 1.8 [1.3–2.5] for emotional neglect, and emotional, physical and sexual violence, respectively). In men, the socio-political context (West Germany) was a predictor of physical and sexual violence (odds ratio [95% confidence interval] 1.8 [1.2–2.7] and 2.5 [1.4–4.5]), respectively. Concerning emotional neglect and violence, socio-political context was not a significant predictor in men. The examination of differential item functioning revealed that our results could not be attributed to differential response behaviour between East and West Germans caused by item bias.

**Conclusions:**

The results suggest that socio-political context is an important determinant to consider when examining childhood maltreatment. Future research should continue to focus on risk and protective factors at the societal level, such as legal frameworks addressing gender equality and child protection laws, to create further evidence for population-wide prevention strategies ensuring safe, nurturing and thriving environments for children and families.

## Introduction

Childhood maltreatment is a widespread phenomenon that can have drastic short and long-term consequences for many aspects of child development (van Harmelen *et al*., 2010; Brydges and Reddaway, 2020), long-term adverse health effects (e.g. Bentley and Widom, 2009; Hemmingsson *et al*., 2014), as well as a diminished economic prospect in adult life (Currie and Spatz Widom, 2010). It constitutes a major risk factor for mental illness (Yanos *et al*., 2010; Teicher *et al*., 2016; Gardner *et al*., 2019; Humphreys *et al*., 2020). On a global scale, there is substantial variation in prevalence rates of self-reported childhood maltreatment in individual studies (Stoltenborgh *et al*., 2012, 2013*a*; Glaesmer, 2016). To unravel this vast variation, the cultural-geographical context was examined as a risk factor; procedural and methodological factors seem to mostly explain the differences in prevalence rates (Stoltenborgh *et al*., 2013*b*). Recent evidence indicates that policy interventions at the socio-political level may be effective in reducing the rates of childhood maltreatment (Austin *et al*., 2020), indicating that socio-political context is either a potential protective factor or risk factor, depending on the economic, social and political framework for child rearing.

### Social-political risk and protective factors for childhood maltreatment

According to the socio-ecological model of childhood maltreatment, there are different levels of risk factors for childhood maltreatment; the individual and interpersonal level, the community level and the societal level (Austin *et al*., 2020). Previous studies have largely focused on the individual and interpersonal level (Austin *et al*., 2020); however, prior studies at the societal level suggest that economic factors (Schneider *et al*., 2007; Brooks-Gunn *et al*., 2013; McLaughlin, 2017), factors relating to gender inequality (Klevens and Ports, 2017; Klevens *et al*., 2018) and political factors (i.e. child protective laws, Clemens *et al*., 2020) are associated with childhood maltreatment. Specifically, economic uncertainty during the great recession has been shown to have a direct effect on the rates of childhood abuse and neglect, independent of individual-level factors (Schneider *et al*., 2007). Gender inequity indices have been found to be associated with the rates of childhood physical abuse and neglect in across-country analyses, after controlling for country development (Klevens and Ports, 2017). Furthermore, legal reforms, such as eliminating corporal punishment of children in Sweden, led to dramatic decline of violent acts against children over recent decades (Durrant and Janson, 2005), indicating child protection laws as a protective factor.

### Socio-political context in East and West Germany

German people experienced political division after World War 2, up until the German reunification. East Germany and West Germany were two socio-political systems with distinct economic, gender and legal norms and, consequently, different approaches towards child rearing. Therefore, one can suspect different risk and protective factors in East and West Germany. Regarding economic factors, a lower economic status has been suggested as a risk factor for childhood maltreatment on the individual level (Euser *et al*., 2011). Economic disparity and precarity have been identified as societal-level risk factors (Brooks-Gunn *et al*., 2013; WHO, 2013). The per capita income before the German reunification was higher in West Germany than East Germany, but basic food and needs were made affordable within East Germany (Lorke, 2015). This may have contributed to economically less precarious conditions and less disparity in East Germany in comparison to West Germany.

Concerning gender equality, both constitutions included a section on gender equality in 1949, but in East Germany, the state enforced equal rights for men and women (Brückner *et al*., 2020), including the right to work, by creating the necessary conditions to make it happen. This implied an out-of-home care in day nursery from early childhood on (Berth *et al*., 2010). In West Germany, few places for out-of-home care facilities/nurseries were available, and it was not until 1996 that local communities were required by law to provide day care for children between 3 and 6 years old (Kreyenfeld and Hank, 1999), possibly contributing to greater gender inequity. Furthermore, in West Germany, individual socio-economic and family factors had a stronger impact on child development in comparison to East Germany, where state-directed policies had a greater effect on shaping institutions of socialisation, emphasising socialist educational goals (Brückner *et al*., 2020). Thus, the upbringing of children in East Germany relied more on collective institutions, a circumstance that may have served as a protective factor against maltreatment in the family, as the embeddedness of child rearing in social networks has been suggested as a protective factor (Korbin, 1991).

In regards to legal norms towards child protection, in East Germany, corporal punishment of children was legally prohibited since its foundation in 1949 (Geißler, 2011). In contrast, in West Germany, it was legally permissible in all schools until 1973 (in Bavaria even until the 80s by ruling of its state supreme court), and only fully abandoned as a legitimate educational measure in 2000 (Fegert *et al*., 2013). Concerning physical violence, a greater acceptance of physical discipline strategies has been shown to relate to a greater use of physical violence (Lansford *et al*., 2010), suggesting a higher risk in West Germany where legal norms may have contributed to a greater normalisation of corporal punishment and an authoritarian educational style (Decker *et al*., 2008; Von Klitzing, 2008). In East Germany, the role of the state gave rise to state-perpetrated institutional violence in orphanages and youth work courts. Due to the overall small number of affected children however, on an epidemiological level, this can be considered statistically not relevant and will not be examined in the current study.

There is a scarcity of studies that consider the German socio-political context as a predictor of the different dimensions of childhood maltreatment. We therefore examined the socio-political context as a predictor of the different dimensions of self-rated childhood maltreatment in a representative sample of the German population. Based on the existing evidence as outlined above and our own unpublished findings in two community samples of East and West Germany, we hypothesised that West Germans would report childhood maltreatment more frequently than East Germans.

## Methods

### Study sample

The data for this analysis consisted of three randomly generated representative samples, derived from face-to-face household surveys of German native speakers above 13 years of age. The surveys were conducted in 2010, 2013 and 2016 by an independent market and social research institute for opinion and social research in Berlin, Germany (USUMA) on behalf of Leipzig University. All three surveys were drawn using a three-stage random sampling procedure, with constituencies (sample points) in the first stage, households in the second (random-route-methods), and individuals within the target households at the third stage (Swedish key procedure). The surveys adhered to the ethical guidelines of the International Code of Marketing and Social Research Practice of the International Chamber of Commerce and of the European Society of Opinion and Marketing Research; for further details of the data assessment see Schilling *et al*. (2016), Beutel *et al*. (2017) and Witt *et al*. (2017). Of 13 964 households (4455, 4607 and 4902, respectively), 7522 households participated (2504, 2508, and 2510, respectively). Of those, participants, with permanent residence, who were born in 1980 or before (*N* = 5905) were selected for the current study, ensuring that the majority of their childhood was spent before German reunification. Socio-political context was defined by geographic location; participants were assigned to either the East or West German group based on state (permanent residence). This method did not allow for the identification of internal migrants, which have been estimated to constitute around 5% of the population for German east-to-west migrants and around 1% of the population for west-to-east migrants (Heller *et al*., 2020). Furthermore, since the data were derived from area samples that considered the size of sampling points, and since the data collection followed pps-design (probabilities proportional to size), East German states, which are less populated than West German states, contained fewer study participants. For data analyses, the final sample comprised 5836 participants, due to the exclusion of participants with missing data regarding child maltreatment (*n* = 69). Socio-demographic characteristics of the study sample in comparison to population data are presented in online Supplementary Table S1. All participants gave informed written consent.

### Assessment of childhood maltreatment

In two surveys (2010 and 2016), the subjection to childhood maltreatment was assessed with the German version of the Childhood Trauma Questionnaire (CTQ), a self-reporting 28-item questionnaire with good psychometric properties (Bernstein *et al*., 2003; Klinitzke *et al*., 2012), while in one survey (2013), the five-item screening version of the CTQ, the Childhood Trauma Screener (CTS; Grabe *et al*., 2012) was used, a reliable and valid screening instrument for the assessment of childhood maltreatment in large population-based studies (Grabe *et al*., 2012). Each of the five CTS items represents one of the five domains of the CTQ, including the following trauma categories: emotional neglect (*when growing up, I felt loved*), physical neglect (*…, there was someone to take me to the doctor if I needed it*), emotional violence (*…, I felt that someone in my family hated me*), physical violence (*…, people in my family hit me so hard, that it left me with bruises or marks*) and sexual violence (*…, someone molested me*). The CTQ and the CTS use a five-point Likert scale (0 = never true; 1 = rarely true; 2 = sometimes true; 3 = often true; 4 = very often true).

### Assessment of depression: Patient Health Questionnaire-2 (PHQ-2)

The PHQ-2 is a short screening questionnaire to assess the core symptoms of major depression (depressed mood and loss of interest or pleasure). The two symptoms are assessed using a four-point Likert scale (‘not at all’ = 0 to ‘almost every day’ = 3); thus the sum score varies between 0 and 6. The screener has sufficient validity and reliability (Kroenke *et al*., 2003; Löwe *et al*., 2005). The PHQ-2 was included in the analyses as an external validation criterion of the binarised CTS items, following Glaesmer *et al*. (2013).

### Statistical analysis

First, we determined the sample characteristics in the sex-stratified sample, and in sex-stratified East and West German samples. We conducted Kolmogorov–Smirnov tests, Mann–Whitney *U*-tests or *T*-tests, to detect differences in age and years of education, household income and depression between East and West German women and East and West German men. Next, we harmonised the surveys by selecting the five CTS items of the CTQ in the 2010 and 2016 surveys. Thereafter we examined their psychometric properties by conducting correlation analyses between the CTS items and the corresponding CTQ dimensions in the East and the West German sample in participants with available CTQ (*n* = 3048; online Supplementary Table S2). In both samples, we found high and significant correlations between the CTS items and the corresponding CTQ dimensions, except for physical neglect in the East German sample where correlations were moderate (online Supplementary Table S2). Thus, we decided to drop the item physical neglect in the main analysis. To assess the internal consistency, we calculated Cronbach's *α* of CTS items emotional neglect, emotional violence, physical violence and sexual violence. The resulting scale reliability coefficient was 0.682, indicating a lower *α* coefficient than the previously reported *α* coefficient (0.765) by Grabe *et al*. (2012).

Following Glaesmer *et al*.'s approach (2013) we transformed each CTS item into a binary variable in the pooled dataset, after reversing the items corresponding to emotional neglect and applying the threshold for individual CTS items (Glaesmer *et al*., 2013). The threshold for emotional neglect was set to  ≥ 3 (3 = often true; 4 = very often true), for physical and emotional violence to  ≥ 2 (2 = sometimes true; 3 = often true; 4 = very often true), and for sexual violence to  ≥ 1 (1 = rarely true; 2 = sometimes true; 3 = often true; 4 = very often true). To identify whether there are sex-specific patterns in either sample, we examined the sex-stratified frequencies of the binarised maltreatment variables in East and West Germany. In the main analysis, logistic sex-stratified regression analyses were used with the four binary outcomes (emotional neglect, emotional violence, physical violence and sexual violence), and socio-demographic context (East *v*. West) as a predictor, without and with covariates (age, education, household income, depression score). All covariates were rescaled to range from 0 (min) to 1 (max), allowing for a comparison of the maximum difference each factor can make. Missing values (<5%) were handled by listwise deletion. To assess if responses differed between participants from East and West Germany due to item bias, we conducted differential item functioning (DIF) analysis. For DIF detection, we used statistical modelling based on an ordinal logistic regression approach (Slocum *et al*., 2004). Differences of Nagelkerke's Pseudo *R*^2^ values reflect effect size measures and were used to identify DIF. Values were interpreted as follows: 0.000 < 0.035 indicated negligible DIF; 0.035 < 0.070 indicated moderate DIF; >0.070 indicated large DIF (Jodoin and Gierl, 2001). All analyses were conducted in Stata (version 16 and 17; StataCorp., 2019, 2021) and R 3.5.2 (R Core Team, 2019). For all tests, the significance level was set at *p* = 0.05. The Benjamini and Hochberg (1995) corrected significance level (logistic regression analyses) was set to *p* = 0.0375 (two-tailed).

## Results

### Sample description

The characteristics of the total sample, and the East and West German subsamples are presented in Table 1. Concurrent with population data, there were more women than men in the sample. The frequency distributions (women/men × East/West) did not differ significantly (*p* = 0.149). Of the 3146 women, 86.1% had turned 18 at the time of the German reunification, while this was the case for 85.1% of the 2690 men. East German women were older (*Z* = 6.622; *p* < 0.001) and had a higher level of formal education (*Z* = 7.647; *p* < 0.001) than West German women. The same was true for East German men, they were older (*Z* = 4.693; *p* < 0.001) and had a higher level of formal education (*Z* = 7.909; *p* < 0.001) than West German men (Table 1). Concerning household income, West German women reported a higher household income than East German women (*Z* = −9.069; *p* < 0.001), as did West German men in comparison to East German men (*Z* = −9.666; *p* < 0.001). In regards to depression scores, no significant differences between East and West German women could be obtained (*p* = 0.403), however, East German men reported higher scores than West German men (*Z* = 2.976; *p* = 0.003).

**Table 1.** Characteristics of the sex-stratified total sample (*N* = 5836) and sex-stratified subsamples

	Total		East Germany		West Germany	
	Women	Men	Women	Men	Women	Men
*n*(%)	3146 (53.9)	2690 (46.1)	655 (52.1)	602 (47.9)	2491 (54.4)	2088 (45.6)
Age						
0.05 percentile	35	35	35	35	35	35
Mean	56.7	56.1	59.9	58.3	55.9	55.4
Median	56	55	61	59	55	55
0.95 percentile	81	79	81	80	81	79
*n* (%)						
Education						
No degree, <8 yrs	72 (2.3)^a^	62 (2.3)^b^	9 (1.4)	11 (1.8)	63 (2.5)	51 (2.5)
Secondary, 8 yrs	1343 (42.7)	1087 (40.5)	203 (29.6)	150 (24.9)	1149 (46.2)	937 (45.0)
Secondary, 10 yrs	1179 (37.5)	905 (33.7)	307 (46.9)	263 (43.7)	872 (35.1)	642 (30.8)
IB equivalent	320 (10.2)	322 (12.0)	69 (10.5)	82 (13.6)	251 (10.1)	240 (11.5)
University degree	228 (7.3)	309 (11.5)	76 (11.6)	96 (16.0)	152 (6.1)	213 (10.2)
Household income						
<500€	13 (0.4)^c^	32 (1.2)^d^	9 (1.4)	15 (2.6)	4 (0.2)	17 (0.8)
500 up to 650€	26 (0.9)	25 (1.0)	12 (1.9)	13 (2.2)	14 (0.6)	12 (0.6)
650 up to 750€	47 (1.6)	31 (1.2)	15 (2.4)	9 (1.5)	32 (1.3)	22 (1.1)
750 up to 900€	106 (3.5)	49 (1.9)	30 (4.8)	17 (2.9)	76 (3.2)	32 (1.6)
900 up to 1000€	123 (4.1)	67 (2.6)	27 (4.3)	25 (4.3)	96 (4.0)	42 (2.1)
1000 up to 1150€	172 (5.7)	67 (2.6)	48 (7.6)	22 (3.8)	124 (5.2)	45 (2.2)
1150 up to 1250€	213 (7.0)	125 (4.8)	47 (7.4)	32 (5.5)	166 (6.9)	93 (4.6)
1250 up to 1500€	396 (13.1)	260 (10.0)	106 (16.8)	70 (12.0)	290 (12.1)	190 (9.4)
1500 up to 2000€	518 (17.1)	501 (19.2)	132 (20.9)	144 (24.7)	386 (16.1)	357 (17.6)
2000 up to 2500€	517 (17.1)	489 (18.7)	108 (17.1)	100 (17.1)	409 (17.0)	389 (19.2)
2500 up to 3500€	533 (17.6)	580 (22.2)	71 (11.2)	110 (18.8)	462 (19.2)	470 (23.2)
3500 up to 5000€	286 (9.4)	286 (11.0)	23 (3.6)	22 (3.8)	263 (11.0)	264 (13.0)
5000€ or more	83 (2.7)	97 (3.7)	4 (0.6)	5 (0.9)	79 (3.3)	92 (4.5)
Mean (s.d.) Depression						
PHQ-2 score	0.92 (1.2)^e^	0.75 (1.1)^f^	0.96 (1.3)	0.86 (1.2)	0.91 (1.2)	0.72 (1.1)

IB, International Baccalaureate; PHQ, Patient Health Questionnaire.

Annotations: ^a^data available for *n* = 3142; ^b^data available for *n* = 2685; ^c^data available for *n* = 3033; ^d^data available for *n* = 2609; ^e^data available for *n* = 3125; ^f^data available for *n* = 2673.

### Description of patterns between women and men in West and East Germany

The frequency distributions of the different maltreatment categories are presented in online Supplementary Table S3. In the West German sample, looking at the binarised neglect item between the sexes, emotional neglect was reported more frequently by women (8.9%) than men (7.1%; *χ*^2^ = 4.878; *p* = 0.027). In contrast, in the East German sample, emotional neglect was more frequently reported by men (8.5%) than women (5.5%; *χ*^2^ = 4.311; *p* = 0.038). Emotional violence was more often reported by women than men in the West German sample (women: 10.1%, men: 7.2%; *χ*^2^ = 11.446; *p* = 0.001) but was similar in the East German sample (women: 6.7%, men: 6.5%, ns). Further, physical violence was more frequently reported by men than women in the East German sample (women: 3.4%, men: 6.0%; *χ*^2^ = 4.897; *p* = 0.027), as well as in the West German sample (women: 8.3%, men 9.9%), where differences were non-significant. Concerning sexual violence, the patterns were similar in both samples – more frequently reported by women (West: women: 13.2%, men 5.4%; *χ*^2^ = 78.500; *p* < 0.001; East: women: 7.8%, men: 2.4%; *χ*^2^ = 19.076; *p* < 0.001), however, the differences were more pronounced in West Germany (online Supplementary Table S3). Since the patterns between the sexes were not the same in both samples, we continued to conduct the following main analyses stratified by sex.

### Socio-political context as a predictor of child maltreatment

**Fig. 1.**
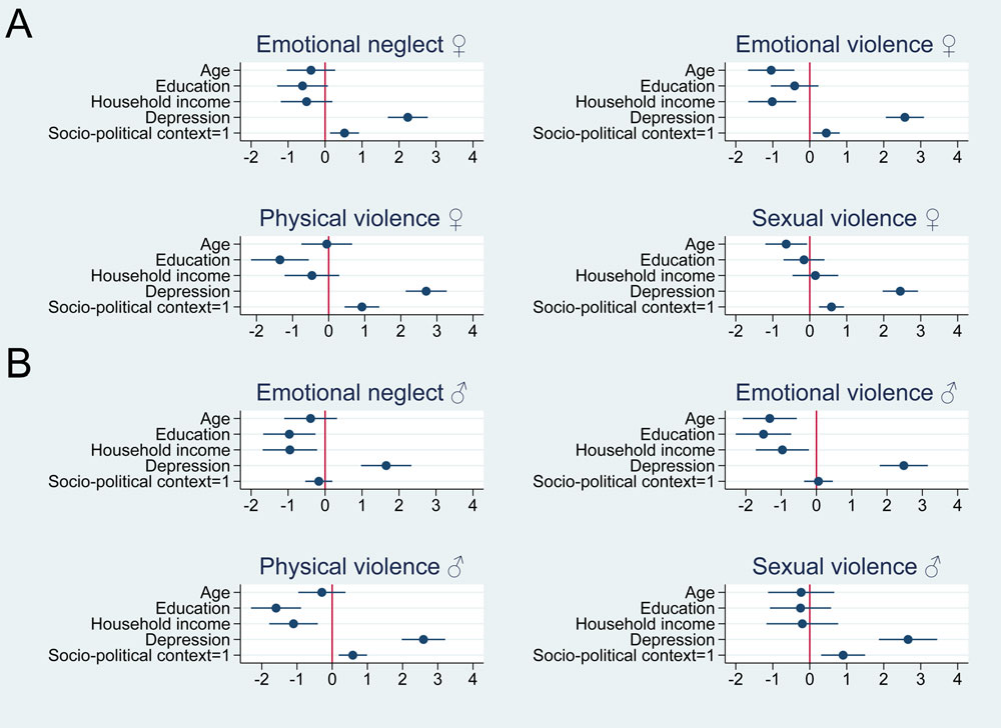
Logistic regression models (*x*-axis: log odds) with socio-political context (East = 0, West = 1) as predictor variable and Childhood Trauma Screener items emotional neglect, emotional violence, physical violence, sexual violence (not present = 0, present = 1) as binary outcomes – including covariates age, education, household income, PHQ-2 sum score – in women (A; *n* = 3009) and men (B; *n* = 2588). Please note that all covariates were rescaled to range from 0(min) to 1(max), allowing for a comparison of the maximum difference each factor can make. PHQ, Patient Health Questionnaire.

The results of the logistic regression analyses are presented in Fig. 1*A* and *B*, and online Supplementary Table S4. As expected, socio-political context (West = 1) was a predictor for all dimensions of child maltreatment in women (Fig. 1*A*, online Supplementary Table S4). Being from West Germany, not East Germany, increased the odds of experiencing emotional neglect and emotional violence, as well as physical and sexual violence, by 1.7 (95% CI 1.16–2.41), 1.6 (95% CI 1.12–2.17), 2.6 (95% CI 1.67–4.08) and 1.8 (95% CI 1.32–2.45) times, respectively (online Supplementary Table S4). These results did not change in principle, when adding age, education, household income and depression scores to the model; here the odds ratios (OR) were 1.7 (95% CI 1.15–2.50), 1.6 (95% CI 1.09–2.25), 2.5 (95% CI 1.56–4.07) and 1.8 (95% CI 1.29–2.52), respectively (Fig. 1*A*, online Supplementary Table S4).

Partially in line with our hypothesis, in men, socio-political context (West = 1) was a significant predictor in logistic regression analyses for having experienced childhood physical and sexual violence (Fig. 1*B*, online Supplementary Table S4), however no association could be obtained regarding emotional neglect and emotional violence (Fig. 1*B*, online Supplementary Table S4). Being from West Germany, not East Germany, was associated with significantly higher odds of experiencing physical violence (OR = 1.7, 95% CI 1.20–2.50) and sexual violence (OR = 2.4, 95% CI 1.37–4.22). When age and education were added into the model, the results remained robust (physical violence OR = 1.8, 95% CI 1.20–2.69; sexual violence OR = 2.5, 95% CI 1.36–4.47; Figure 1*B*, online Supplementary Table S4).

### DIF analysis

Finally, we conducted DIF analyses of CTS items to detect potential item bias in response behaviour between participants from East and West Germany. Analysis was directed to investigate uniform as well as non-uniform DIF using ordinal logistic regression. Results indicate that none of the items showed DIF between both groups. The values of the effect size (Nagelkerke's Pseudo *R*^2^ differences) for the CSQ items ranged between 0.000 and 0.005 for uniform DIF and 0.004 and 0.011 for non-uniform DIF. Thus, all effect sizes were substantially below the critical value of 0.035 (online Supplementary Table S5).

## Discussion

In the current study, we examined whether socio-political context predicts child maltreatment in women and men, as assessed by the screening version of the CTQ. In women, we found that socio-political context (West Germany) predicted all examined types of childhood maltreatment, as hypothesised. There was a higher risk of experiencing emotional neglect, emotional, physical and sexual violence for participants from West Germany compared to East Germany. In men, the socio-political context predicted the experience of physical and sexual violence, indicating that there was a higher risk of experiencing physical and sexual violence for West German participants, compared to East German participants. Contrary to our expectations, socio-political context did not predict emotional neglect and violence in men. Overall, effect sizes were small to moderate. Our results could not be attributed to differences in response behaviour.

Among the different dimensions of childhood maltreatment, socio-political context was the strongest predictor for the experience of physical and sexual violence in women and men. Concerning physical violence, it is conceivable that this is due to the different societal norms and the legal frameworks in West Germany *v*. East Germany. Corporal punishment was legally permissible up to the late 20th century in former West Germany (Decker *et al*., 2008; Von Klitzing, 2008), whereas it was banned in 1949 in East Germany, with the founding of the former East German state (Geißler, 2011). Different policies and definitions of physical maltreatment have been shown to influence its rates (Gilbert *et al*., 2012), which may explain the higher rates of self-reported physical maltreatment in West Germany. This view is corroborated by the dramatic decline of corporal punishment in Sweden, after its ban as an educational measure in 1979 (Durrant and Janson, 2005), and similarly by the decrease of corporal punishment in Germany as a whole, after the legal enforcement of children's right to an upbringing without violence in the German Civil Code in 2000 (Clemens *et al*., 2020).

Concerning sexual violence in women, our results are in line with an earlier study by Schötensack *et al*. (1992), who described higher rates of childhood sexual abuse in a West German community sample, in comparison to a community sample in East Germany (West: 16.1%; East: 9.6%), however, frequencies in the current study were slightly lower (West: 13.2%, East: 7.8%) possibly due to differences in sample selection (regional *v*. population-based). Concerning sexual violence in men, Schötensack *et al*. (1992) reported no differences between East and West German men (5.8% in both samples), in contrast to our findings of 5.4% in the West German sample and 2.4% in the East German sample, which again could be related to differences in sample selection.

Our findings can be discussed against the background of aforementioned risk factors. One can speculate that the greater equality of women and men in East Germany contributed to the lower rates of sexual violence, leading to a smaller structural power gradient between women and men. In East Germany, both women and men had the same legal status in all areas of social, state and personal life (GDR, 1949), thus women had a basic right to work (Sackmann *et al*., 2013). Furthermore, in 1972, abortions were ruled legal and seen as a necessary step to achieve gender equality (Günther, 1992). These legal rulings led to an empowerment of women and girls in East Germany, reducing strong hierarchical models that were still prevalent in West Germany, where married women were often financially dependent on their spouses. It has been postulated that the society in East Germany had greater gender equality in employment and income (Stöbel-Richter *et al*., 2012; Lüschen *et al*., 1997), even though women were often carrying the double load, as mother and worker, and often had ‘female jobs’ (Nickel, 1990). In contrast, most West German women were housewives or only part-time workers (Spellerberg, 1996) and were often not equally paid. This greater structural gradient of equality between women and men may have rendered children generally more vulnerable to sexual violence in former West Germany, as gender inequality and inequity are known risk factors for sexual violence during childhood (Jones and Trotman Jemmott, 2016; Klevens and Ports, 2017; Klevens *et al*., 2018). Moreover, while children in East Germany were under the surveillance and care of state institutions (e.g. day care, after school, youth organisations), the West German postwar society relied predominantly on the nuclear family for child rearing, making it difficult to detect child maltreatment in risky parental constellations. Thus, the greater institutional care in East Germany might have contributed to an earlier detection of violent behaviour towards children in the family. Another possible explanation may be a different response behaviour between East and West Germans. Our sensitivity analysis could, however, not detect any differences in response behaviour between East and West Germans.

Concerning emotional neglect and violence, here we found different patterns in women and men. While the higher odds for emotional neglect and violence in West German women may be indicative of the above-mentioned differences in gender policies, the odds for emotional neglect and violence between West German and East German men were similar and may point to lower emotional awareness in men in general (Barrett *et al*., 2000).

We were not able to include the physical neglect item due to its psychometric property, which has been shown to lack validity in several studies (Bernstein *et al*., 2003; Grabe *et al*., 2012; Klinitzke *et al*., 2012; Glaesmer *et al*., 2013). It has been suggested that the dimension of physical neglect may particularly be affected by cultural, historical and socio-economic influences, making it difficult to interpret it over time (Glaesmer *et al*., 2013). For example, for older participants who grew up in the post-war period, a general scarcity of material resources and medical care structures, e.g. not being brought to the doctor when necessary, may have not been a sign of neglect, but simply a sign of the lack of adequate infrastructure.

Other limitations of the study include the self-rated assessment of childhood maltreatment, which did not include state-perpetrated and institutional violence. Further, due to the sampling procedure and the representative nature of the surveys, the East German sample contained fewer study participants, possibly creating a bias in the binary CTS outcomes with a smaller number of events. To mitigate the problem, we used a large sample size to avoid small sample size bias in the logistic regression analyses. However, concerning sexual abuse in East German men, there was only a small number of events, possibly resulting in bias. Recalculating the model using penalised likelihood indicated that the results were robust. Moreover, we were not able to identify internal migrants, since the group assignment (East, West German) was based on geographic location (state of permanent residency). We estimated the relative frequencies of internal migrants compared to the total sample based on published data (east-to-west migrants: around 5% of the population; west-to-east migrants: around 1% of the population; Heller *et al*., 2020). Based on these numbers and reported prevalence rates of child maltreatment in East and West Germany, the omission of internal migrants is negligible.

Findings may have been attributed to differences in response behaviour between East and West Germans, however we could not detect potential item bias in response behaviour between participants from East and West Germany. Strengths include the sample size, allowing for sex-stratified analyses, the use of representative data and the unique study setting.

To conclude, our findings support the thesis that socio-political context is an important determinant to consider, when examining childhood maltreatment. Future studies should continue to address factors at the societal level, such as legal frameworks addressing gender equality and child protection laws, to create evidence for population-wide prevention strategies ensuring safe, nurturing and thriving environments for children and families.

## Data Availability

The data are available from the authors upon request.

## Supplementary material

For supplementary material accompanying this paper visit http://dx.doi.org/10.1017/S2045796021000585. click here to view supplementary material
