# Supplementary material for: Socio-political context as determinant of childhood maltreatment: a population-based study among women and men in East and West Germany
Source: Epidemiol Psychiatr Sci. 2021 Nov 18;30:e72. doi: 10.1017/S2045796021000585 (PMC8611929; doi:10.1017/S2045796021000585)
Supplement: Supplementary file 1 [file S2045796021000585sup001.docx]

**Supplementary materials**

**Table S1.** **Socio-demographic characteristics of the study sample.**

|  |  | **Total population**  **of Germany [%]** | | **Sample**  ***N*=5860 [%]** |
| --- | --- | --- | --- | --- |
| **Gender** | | | | |
|  | Male | 48.6 | | 46.1 |
|  | Female | 51.4 | | 53.9 |
|  | Diverse | n/a | | n/a |
| **Age** | | | | |
|  | 18-25 | 11.3 | | - |
|  | 26-45 | 31.9 | | 26.2 |
|  | 46-60 | 26.9 | | 34.7 |
|  | >60 | 29.9 | | 39.1 |
| **Education** | | | | |
| No schooling completed/unknown | | | 5.0 | 2.3 |
|  | ≤ 9 y | 38.5 | | 41.7 |
|  | 10 y | 29.3 | | 35.8 |
|  | >10 y | 27.1 | | 20.2 |

Caption: Population data from the Federal Office of Statistics (Dec. 2010)

**Table S2. Bivariate Pearson correlations between the items of the Childhood Trauma Screener (CTS), respective Childhood Trauma Questionnaire (CTQ) dimensions and their sum scores. Data based on participants living in former East-German (*n*=825) und West-German states (*n*=3048).**

|  | **CTQ** dimensions | | | | | |  |
| --- | --- | --- | --- | --- | --- | --- | --- |
|  | **emotional abuse** | **physical abuse** | **sexual abuse** | **emotional neglect** | **physical neglect** | **CTQ-sum** |  |
| **East** |  |  |  |  |  |  |  |
| CTS Items |  |  |  |  |  |  |  |
| emotional abuse | **0.744***** | 0.515*** | 0.294*** | 0.369*** | 0.314*** | 0.600*** | |
| physical abuse | 0.561*** | **0.844***** | 0.207*** | 0.347*** | 0.291*** | 0.590*** | |
| sexual abuse | 0.261*** | 0.239*** | **0.816***** | 0.174*** | 0.127*** | 0.336*** | |
| emotional neglect | 0.416*** | 0.395*** | 0.215*** | **0.735***** | 0.410*** | 0.665*** | |
| physical neglect | 0.122*** | 0.118** | 0.032 ns | 0.431*** | **0.631***** | 0.444*** | |
| CTS-sum | 0.596*** | 0.580*** | 0.340*** | 0.728*** | 0.677*** | **0.847***** |  |
| **West** |  |  |  |  |  |  |  |
| CTS Items |  |  |  |  |  |  |  |
| emotional abuse | **0.775***** | 0.553*** | 0.403*** | 0.360*** | 0.371*** | 0.624*** |  |
| physical abuse | 0.564*** | **0.842***** | 0.415*** | 0.351*** | 0.427*** | 0.643*** |  |
| sexual abuse | 0.433*** | 0.415*** | **0.888***** | 0.224*** | 0.264*** | 0.510*** |  |
| emotional neglect | 0.452*** | 0.358*** | 0.236*** | **0.811***** | 0.525*** | 0.688*** |  |
| physical neglect | 0.224*** | 0.237*** | 0.161*** | 0.509*** | **0.700***** | 0.521*** |  |
| CTS-sum | 0.674*** | 0.652*** | 0.524*** | 0.743*** | 0.521*** | **0.890***** |  |

**Table S3. Item frequencies (Childhood Trauma Screener) in the stratified samples.** Each of the items (1=emotional neglect, 2=emotional violence, 3=physical violence, 4=sexual violence) are scored on a 5-point Likert scale (1=never true, 2=rarely true, 3=sometimes true, 4=often true, 5=very often true). Data based on participants (born 1980 or earlier) living in former East-German (*n*=1257) and West-German states (*n*=4579). White/grey fields indicate relevant thresholds for dichotomization (white=0, grey=1), according to Glaesmer et al. (2013).

| **Women** | **East, *n* (%)** | | |  |  |  | **West, *n* (%)** | |  |  |  |
| --- | --- | --- | --- | --- | --- | --- | --- | --- | --- | --- | --- |
| **When growing up…** | **Never true** | **Rarely true** | **Sometimes true** | **Often true** | **Very often true** |  | **Never true** | **Rarely true** | **Sometimes true** | **Often true** | **Very**  **often true** |
| 1. I felt loved. | 12  (1.8) | 24  (3.7) | 61  (9.3) | 223  (34.0) | 335  (51.1) |  | 84 (3.4) | 137  (5.5) | 267  (10.7) | 870  (34.9) | 1133  (45.5) |
| 2. I felt that someone in my family hated me. | 568  (86.7) | 43  (6.6) | 25  (3.8) | 16  (2.4) | 3  (0.5) |  | 1998  (80.2) | 242  (9.7) | 156  (6.3) | 67  (2.7) | 28  (1.1) |
| 3. People in my family hit me so hard that it left me with bruises or marks. | 591  (90.2) | 42  (6.4) | 14  (2.1) | 5  (0.8) | 3  (0.5) |  | 2041 (81.9) | 243  (9.8) | 132  (5.3) | 54  (2.2) | 21  (0.8) |
| 4. Someone molested me. | 604  (92.2) | 33 (5.0) | 12  (1.8) | 6  (0.9) | - |  | 2163  (86.8) | 177  (7.1) | 111  (4.5) | 23  (0.9) | 17  (0.7) |
| **Men** | **East, *n* (%)** | |  |  |  |  | **West, *n* (%)** | |  |  |  |
| 1. I felt loved. | 18  (3.0) | 33  (5.5) | 67  (11.1) | 237  (39.4) | 247  (41.0) |  | 50 (2.4) | 98  (4.7) | 265  (12.7) | 863  (41.3) | 812  (38.9) |
| 2. People in my family hit me so hard that it left me with bruises or marks. | 508  (84.4) | 58  (9.6) | 25  (4.2) | 8  (1.3) | 3  (0.5) |  | 1637 (78.4) | 244  (11.7) | 138  (6.6) | 53  (2.5) | 16  (0.8) |
| 3. I felt that someone in my family hated me. | 508  (84.4) | 55  (9.1) | 21  (3.5) | 13  (2.2) | 5  (0.8) |  | 1698 (81.3) | 239  (11.4) | 95  (4.5) | 39  (1.9) | 17  (0.8) |
| 4. Someone molested me. | 588  (97.7) | 13  (2.2) | - | - | 1  (0.2) |  | 1975 (94.6) | 68  (3.3) | 34  (1.6) | 9  (0.4) | 2  (0.1) |

**Table S4.** **Logistic regression models with socio-political context (East=0, West=1) as predictor variable and the Childhood trauma screener items (no=0/yes=1) as binary outcomes, and covariates in women (*n*=3146/3009) and men (*n=*2690/2588*)*.**

| **Women** |  | |  | | **Men** | |
| --- | --- | --- | --- | --- | --- | --- |
| ***Model 1-4*** | ***OR (95%CI low, high)*** | ***SE*** | | ***OR (95%CI low, high)*** | | ***SE*** |
| **Emotional neglect ~ East/West** | 1.674(1.16, 2.41) | 0.31** | | 0.824(0.59, 1.15) | | 0.14 |
| intercept | 0.581(0.04,0.08) | 0.01**** | | 0.093(0.07, 0.12) | | 0.01**** |
| **Emotional violence ~ East/West** | 1.556(1.12, 2.17) | 0.26** | | 1.125(0.78, 1.62) | | 0.21 |
| intercept | 0.072(0.05,0.10) | 0.01**** | | 0.069(0.05-0.10 | | 0.01**** |
| **Physical violence ~ East/West** | 2.608(1.67, 4.08) | 0.60**** | | 1.730(1.20, 2.50) | | 0.32** |
| intercept | 0.035(0.02, 0.05) | 0.01**** | | 0.063(0.05, 0.10) | | 0.01**** |
| **Sexual violence ~ East/West** | 1.796(1.32, 2.45) | 0.28**** | | 2.403(1.37, 4.22) | | 0.69** |
| intercept | 0.084(0.06, 0.11) | 0.01**** | | 0.024(0.01, 0.04) | | 0.01**** |
| ***Model 1a-4a*** |  |  | |  | |  |
| **Emotional neglect ~ East/West** | 1.694(1.15, 2.50) | 0.34** | | 0.845 (0.59, 1.22) | | 0.16 |
| +Age | 0.684(0.36, 1.31) | 0.23 | | 0.677(0.33, 1.38) | | 0.25 |
| +Education | 0.544(0.27, 1.08) | 0.19 | | 0.381(0.19, 0.77) | | 0.14** |
| + Household income | 0.607(0.30, 1.22) | 0.22 | | 0.387(0.19, 0.81) | | 0.22* |
| + Depression | 9.374(5.47, 16.06) | 2.58**** | | 5.22(2.65, 10.31) | | 1.81**** |
| intercept | 0.076(0.04, 0.16) | 0.03**** | | 0.231(0.12, 0.46) | | 0.08**** |
| **Emotional violence ~ East/West** | 1.565(1.09, 2.25) | 0.29* | | 1.059(0.71, 1.59) | | 0.22 |
| +Age | 0.353(0.19, 0.66) | 0.11* | | 0.267(0.12, 0.57) | | 0.10** |
| +Education | 0.665(0.35, 1.27) | 0.22 | | 0.223(0.10, 0.49) | | 0.09**** |
| + Household income | 0.363(0.02, 0.69) | 0.12** | | 0.381(0.18, 0.81) | | 0.15* |
| +Depression | 13.10(7.84, 21.89) | 3.43**** | | 11.881(6.02, 23.44) | | 4.12**** |
| intercept | 0.144(0.07, 0.29) | 0.05**** | | 0.273(0.13, 0.56) | | 0.10**** |
| **Physical violence ~ East/West** | 2.517(1.56, 4.07) | 0.62**** | | 1.798(1.20, 2.69) | | 0.369** |
| +Age | 0.950(0.47, 1.92) | 0.34 | | 0.746(0.38, 1.46) | | 0.26 |
| + Education | 0.259(1.12, 0.58) | 0.11 ** | | 0.203(0.10, 0.41) | | 0.07**** |
| + Household income | 0.630(0.30, 1.34) | 0.24 | | 0.333(0.17, 0.66) | | 0.12** |
| +Depression | 14.9(8.46, 26.32) | 4.32**** | | 13.376(7.22, 24.79) | | 4.21**** |
| intercept | 0.046(0.02, 0.11) | 0.02**** | | 0.175(0.09, 0.35) | | 0.06**** |
| **Sexual violence ~ East/West** | 1.800(1.29, 2.52) | 0.21** | | 2.468(1.36, 4.47) | | 0.75** |
| +Age | 0.529(0.30, 0.93) | 0.15* | | 0.792(0.32, 1.94) | | 0.36 |
| + Education | 0.856(0.49, 1.49) | 0.24 | | 0.780(0.34, 1.78) | | 0.33 |
| + Household income | 1.164(0.63, 2.16) | 0.37 | | 0.818(0.31, 2.16) | | 0.40 |
| +Depression | 11.58(7.18, 18.66) | 2.82**** | | 14.27(6.49, 31.39) | | 5.74**** |
| intercept | 0.645(0.33, 0.125) | 0.02**** | | 0.020(0.01, 0.06) | | 0.01**** |

**Annotations**: East=0, West=1; **p* < .0375 (Benjamini Hochberg correction); ***p* < .01; ****p* < .001; ****p< .0001 (two-sided).

**Table S5.** **Differential Item Functioning (DIF) analysis for uniform and non-uniform DIF of the CTS items between respondents from former East-German (n=1257) and West-German states (n=4579).**

|  | **Uniform DIF** | **Non-Uniform DIF** |
| --- | --- | --- |
|  | *ES (R^2^-Diff)* | *ES (R^2^-Diff)* |
| 1. I felt loved. | 0.005 | 0.005 |
| 2. People in my family hit me so hard that it left me with bruises or marks. | 0.004 | 0.010 |
| 3. I felt that someone in my family hated me. | 0.001 | 0.004 |
| 4. Someone molested me. | 0.004 | 0.011 |
| 5. There was someone to take me to the doctor if I needed it. | 0.000 | 0.004 |

**Annotations:** *ES (R^2^-Diff) = Effect sizes based values of Nagelkerke’s Pseudo-R^2^ differences.*
